# General Pathology

**Published:** 1895-12-14

**Authors:** 


### Progress in Medicine.

### GENERAL PATHOLOGY.

**On the Pathology of Fevers.**—Among the ever-increasing results of bacteriology we may notice that the differentiation of the various forms of organism allied to the b. coli is aided by observing their behaviour in fermentation tubes containing sugar bouillon. T. Smith[1] shows the value of this test taken in conjunction with others, but the quantity of the gas evolved, the rate of production, its composition, and the formation of acid and behaviour with different sugars must be taken into account, as well as the characters of the potato cultures and the indol reaction. Salvati and Gaetano[2] have obtained an antitoxic serum which protected animals from the action of poisonous doses of the b. coli. When both the poison and the antitoxin were given together the animals survived. Akerman[3] tries to show that many cases of osteomyelitis are due to this organism, since injections of it in young rabbits produced abscesses in the long bones near the epiphyses. The means of combating streptococcal diseases are becoming evident. Marmorek, Charrin, and Roger have obtained an antitoxin which is both preventive and curative. Roger[4] reported the successful treatment by it of puerperal septicœmia, erysipelas in a new-born child, and severe tonsillitis during the puerperium; while Marmorek treated forty-five cases of erysipelas by a similar serum. Bokenham[5] finds that the streptococci from erysipelas and from abscesses are not specifically different, but that any type may be obtained by cultivation from a single stock. The great difficulty of growing the streptococcus without loss of its virulence has been overcome by the use of bouillon mixed with asses' serum. In this manner, or by growing in a series of living animals, can the power of the streptococcus be alone kept up. The importance of obtaining a powerful anti-streptococcal serum is very great, as Bokenham points out, not only for diseases due solely to that microbe, but also for the mixed cases of diphtheria, where it is present.

Why the growth of some organisms inside the body is followed by suppuration, and that of others is not productive of it, is not altogether clear. Roswell Park divides[7] pus producing microbes into obligate, or those normally doing so, such as the staphylo and strepto-cocci, the colon bacilli, the m. tetragonus, the b. pyocyaneus, and certain virulent types of pneumococus; and, on the other hand, the faculative types, such as b. tuberculosis and tetani, which do so only occasionally and exceptionally. The work of Babes, Bokenham, and others tends to show that varying virulence of the streptococci may cause abscess, erysipelas, scarlet fever, pyœmia; and though their peptonising (or solvent action) on the tissues is small, they pass along vessels and lymph spaces with marvellous rapidity. Park notices, too, the wide difference between substances commonly called pus, *e.g.*, the excess of formative material on a granulating surface, and true septic pus from an abscess. Some of the pus-producing organisms will not grow in the vicinity of silver, copper, and other metals, as shown in the experiments of Halstead, and this discovery may have a practical application. Finally, the effects of the association of several microbes is not the same in animals of different species, according to Roger, for the action of anthrax on guinea pigs is greatly intensified if the b. prodigiosus is present with the anthrax, while rabbits survive a fatal dose of anthrax if the other microbe is added.[8] Intimately connected with the production of pus is the phenomenon of leucocytosis. Goldscheider and Jacob showed not long ago that hypoleucocytosis is produced by the leucocytes being held in the capillaries, while hyperleucocytosis implies a general increase and not a mere alteration in distribution.[9] The first state is produced after the injection of toxic matters, such as bacterial cultures. Here the blood from the large vessels is denuded of leucocytes, and the capillaries filled with them. It is suggested that this is in favour of the invading bacteria, for very few are at first taken up by the leucocytes, as Metschnikoff's theory of phagocytosis would imply, but rather that they thus obtain a chance of attacking the walls of the vessels. Following this transient diminution or rearrangement which follows an injection of toxic matter there is a true hyperleucocytosis, the object of which is to elaborate some chemical substance[10] which acts as an antitoxin. It is right to add that Ewing[11] and others regard this early disappearance of leucocytes from the arteries and veins as phagocytic in nature, and that the leucocytes absorb the bacteria in the capillaries, and with them are drained off by the liver cells for removal from the body. Loewy and Richter[12] found that artificial hyperleucocytosis prevented the fatal effect of pneumococci injections if it was excited soon enough, and that a febrile temperature produced by puncture of the brain exerted a similar protective influence against moderately severe inoculations. Hence, it would seem that fevers, as well as leucocytosis, are one of nature's means of protection, and that drugs which act on the thermogenic centres tend to increase the virulence of the disease,[13] though quinine may make up for this by the force of its bactericidal action. Cold baths, too, do not affect the centres at all, but prevent the accumulation of heat.

In the discussions on serum treatment we find Klein pointing out[14] that the antitoxic and the germicidal or protecting substances produced are not the same, the former being of the nature of a ferment. Indeed, as Bouchard[15] adds, toxins and antitoxins are being constantly produced by the cells of the healthy body, hemi-albumoses and anti-albumoses, coagulative and anti-coagulative products of the pancreas, and so on, between which a vital equilibrium is maintained. Now, in toxic diseases, where the germs themselves remain localised, the power of increasing the output of a particular antitoxin is all-important; while in septic ones, the germicidal power may be especially needed. It has been shown that serum is naturally germicidal to some degree, possibly from a substance secreted by eosinophile cells; though Mesnil[16] seems to have proved in the case of certain fishes that the power is quite independent of these cells. This defensive secretion has been identified with nuclein, which is soluble in alkalies, so that when an increase of alkalinity of the blood and of the number of the white corpuscles takes place we have at once an increase of the protective power from the nuclein poured out.[17] Possibly the real state of the case is that a general germicide nuclein is produced in varying quantities, and special antitoxins to meet special poisons, both from the body fluids.

We may notice here, too, Delbet advocates treatment of infective diseases by the injection of immunized blood[18] and not of serum, as containing more of the curative agents than serum does. He prevents coagulation by adding oxalate of sodium, and finds it is rapidly absorbed without ill effects. It has been objected to the ordinary serum treatment that such injections of foreign serum must destroy the corpuscles of human blood, but this only occurs when large quantities are injected[19] direct into the veins, not under the conditions of injections as now used. All the facts are opposed to such a view.

**Snake Poisons.**—Various papers appeared early in the year on the Mueller method of treating snake bite by injections of strychnine in large doses, but the results in most instances were unsatisfactory. Indeed, Elliott considers it tends to hasten death.[20] The use of chloride of lime, advocated by Calmette, has also been found by Phisalix and Bertrand[21] to depend only on its local action as a caustic. Hodgson and Mackensie,[22] however, report successful cases where it was injected into various parts of the body, not always where the bite took place. Calmette[23] has by repeated injections of small quantities of serpent poison rendered animals immune to large doses even 80 times the lethal one, while their serum renders other animals immune. Hypochloride of lime or gold chloride neutralise the venom, hence his advice to inject one of them after ligaturing the limb. He also insists that animals immunised against one kind of poison are to ome extent protected against one or more other infective diseases. The virulence of snake poison varies with the length of time since the animal has last eaten or bitten before the venom is collected, and increases with a long fast.[24] The ornithorhynchus[25] is found to have a true poison gland opening on a spur, and producing a venom allied to that of the Australian snakes. A most important advance has been made by Fraser[26] working with the venom of typical snakes of the most poisonous kinds. He not only immunised rabbits to bear an injection of 50 times the minimum lethal dose, but he obtained an anti-venene from them which prevented any ill effects when injected thirty minutes after fatal doses of the poison had been given to otherwise unprotected animals. A horse is being rendered highly immune so that the treatment can be extended to man, and more powerful anti-venene elaborated. It was also discovered that venom can be administered by the stomach in enormous doses without ill-effects,[27] and confers a considerable degree of protection against subsequent subcutaneous injections, which may explain the protection enjoyed by certain snake charmers. Similarly the blood of poisonous snakes contains much anti-venene capable of neutralising some amount of the poison. Contrary to the results found with tetanus, the antagonism between the poison and the antidote appears to be that of a chemical reaction, the most striking results being found when the two are mixed together before injection. This view is supported by the greater resistance made by animals fed on a flesh diet to the effects of venom. Other experiments showed the value of often-repeated injections of anti-venene after a bite, and a supply of it has been already sent to India in a dried form for use against cobra bites.

**Pyrexia.**—Jacobi reported[28] a case where various thermometers marked 148 degrees, and in spite of all precautions no explanations could be discovered. It was pointed out that the temperature of the urine is important in detecting cases of malingering. J. M. Anders[29] finds that guiacol externally not only reduces temperature, sometimes causing a profuse sweating and chill, but allays pain. This analgesic effect is still more marked when injected hypodermically. Sciatica and supraorbital neuralgia were rapidly relieved by injections of two mimims of guiacol in ten of chloroform under the skin.

[1] Am.J.M.Sc., Sep. [2] B.M.J.. Aug. 10. [3] B.M.J., Aug. 17. [4] B.M.J., May 4. [5] B.M.J., June 15. [6] B.M.J., Sep. 14. [7] Med. Rec., June 8. [8] Med. Week, March 24. [9] Am.M.S. Bulletin, July 1. [10] Ind. Med. Chir. Rev., March. [11] N. Med J., March 2. [12] B.M.J., Aug. 3. [13] Med. Rec., Ap. 27. [14] B.M.J., Aug. 17. [15] B.M J., Sep. 14. [16] B.M.J., June 29. [17] Ther. Gazette, Ap. [18] Med. Week, July 8. [19] Ther. Gazette, Aug. 15. [20] B.M.J., Ap. 20. [21] B.M.J., July 27. [22] Lancet, Feb. 9. [23] B.M.J., June 15. [24] B.M.J., Sep. 7. [25] Lancet, July 6. [26] B.M.J., June 15. [27] B.M.J., Aug. 17. [28] Med. Rec., June 15. [29] Ther. Gaz., March 15.

